# Tackling strong biofilm and multi-virulent vancomycin-resistant *Staphylococcus aureus* via natural alkaloid-based porous nanoparticles: perspective towards near future eradication

**DOI:** 10.3389/fcimb.2023.1287426

**Published:** 2024-01-12

**Authors:** Marwa I. Abd El-Hamid, Doaa Ibrahim, Sara T. Elazab, Wafaa M. Gad, Marwa Shalaby, Wafaa M. El-Neshwy, Mohammed Abdulrahman Alshahrani, Ahmed Saif, Reem M. Algendy, Maha AlHarbi, Fayez M. Saleh, Afaf Alharthi, Eman A. A. Mohamed

**Affiliations:** ^1^ Department of Microbiology, Faculty of Veterinary Medicine, Zagazig University, Zagazig, Egypt; ^2^ Department of Nutrition and Clinical Nutrition, Faculty of Veterinary Medicine, Zagazig University, Zagazig, Egypt; ^3^ Department of Pharmacology, Faculty of Veterinary Medicine, Mansoura University, Mansoura, Egypt; ^4^ Department of Bacteriology, Animal Health Research Institute (AHRI), Mansoura Branch, Agriculture Research Center, Mansoura, Egypt; ^5^ Department of Animal Medicine, Infectious Diseases, Faculty of Veterinary Medicine, Zagazig University, Zagazig, Egypt; ^6^ Department of Clinical Laboratory Sciences, Faculty of Applied Medical Sciences, Najran University, Najran, Saudi Arabia; ^7^ Department of Clinical Laboratory Sciences, College of Applied Medical Sciences, King Khalid University, Abha, Saudi Arabia; ^8^ Food Hygiene, Safety and Technology Department, Faculty of Veterinary Medicine, Zagazig University, Zagazig, Egypt; ^9^ Department of Biology, College of Science, Princess Nourah bint Abdulrahman University, Riyadh, Saudi Arabia; ^10^ Department of Medical Microbiology, Faculty of Medicine, University of Tabuk, Tabuk, Saudi Arabia; ^11^ Department of Clinical Laboratory Sciences, College of Applied Medical Sciences, Taif University, Taif, Saudi Arabia

**Keywords:** natural alkaloids, nanoparticles, biofilm, QS, VRSA, multi-virulent, inflammation

## Abstract

**Introduction:**

As a growing direction, nano-based therapy has become a successful paradigm used to address the phytogenic delivery-related problems in overcoming multivirulent vancomycin-resistant *Staphylococcus aureus* (VRSA) infection.

**Methods:**

Hence, our aim was to develop and assess a novel nanocarrier system (mesoporous silica nanoparticles, MPS-NPs) for free berberine (Free-BR) as an antimicrobial alkaloid against strong biofilm-producing and multi-virulent VRSA strains using *in vitro* and *in vivo* mouse model.

**Results and discussion:**

Our outcomes demonstrated vancomycin resistance in 13.7% of *Staphylococcus aureus* (*S. aureus*) strains categorized as VRSA. Notably, strong biofilm formation was observed in 69.2% of VRSA strains that were all positive for *icaA* gene. All strong biofilm-producing VRSA strains harbored a minimum of two virulence genes comprising *clfA* and *icaA* with 44.4% of them possessing all five virulence genes (*icaA*, *tst*, *clfA*, *hla*, and *pvl*), and 88.9% being multi-virulent. The study findings affirmed excellent *in vitro* antimicrobial and antibiofilm properties of BR-loaded MPS-NPs. Real-time quantitative reverse transcription PCR (qRT-PCR) assay displayed the downregulating role of BR-loaded MPS-NPs on strong biofilm-producing and multi-virulent VRSA strains virulence and *agr* genes in both *in vitro* and *in vivo* mice models. Additionally, BR-loaded MPS-NPs supplementation has a promising role in attenuating the upregulated expression of pro-inflammatory cytokines’ genes in VRSA-infected mice with attenuation in pro-apoptotic genes expression resulting in reduced VRSA-induced apoptosis. In essence, the current study recommends the future scope of using BR-loaded MPS-NPs as auspicious alternatives for antimicrobials with tremendous antimicrobial, antibiofilm, anti-quorum sensing (QS), and anti-virulence effectiveness against problematic strong biofilm-producing and multi-virulent VRSA-associated infections.

## 1 Introduction

Infectious bacterial diseases represent one of the substantial causes of morbidity and death worldwide, especially in developing countries. The growing incidence of antimicrobial resistance together with inadequate drug choices make the therapy for various bacterial infections more troublesome (Bialvaei and Samadi Kafil, 2015; Kafil et al., 2016; Aghapour et al., 2019). One of the most serious bacterial species developing a multidrug-resistance (MDR) phenomenon worldwide is *Staphylococcus aureus* (*S. aureus*), particularly methicillin-resistant *Staphylococcus aureus* (MRSA) (Ammar et al., 2015). It constitutes a life-threatening bacterial pathogen causing serious community, nosocomial, foodborne (Elmowalid et al., 2022) and animal infections including pneumonia, cutaneous infections, septic arthritis, endocarditis, and mastitis. This situation has recently intensified with the emergence of vancomycin-resistant *S. aureus* (VRSA) (Dhanalakshmi et al., 2012). The success of this bacterial pathogen to avoid the impact of antimicrobial agents and host immune response (Van Acker et al., 2014) is accomplished through its antimicrobial resistance in addition to the possession of many virulence related genes. The higher severity of VRSA infections is attributable to its production of numerous toxins and biofilm-forming aptitude, which facilitates its persistence and renders the microorganisms more resistant with immune evasion characteristics (Tristan et al., 2003; Laverty et al., 2013; Ren et al., 2020). Staphylococcal virulence factors including biofilm capability are controlled, to a large extent, by cell-to-cell communication quorum sensing (QS), which is known as an accessory gene regulator (*agr*) system (Le and Otto, 2015). Based on the vital role of QS in the regulation of VRSA virulence and biofilm, numerous strategies have been established targeting this signaling system (Jiang et al., 2019; Abd El-Hamid et al., 2020). Therefore, there is a robust necessity for other novel antimicrobials with anti-QS properties to monitor serious issues related to bacterial virulence and antibiotic resistance spread (Barros et al., 2009; Ibrahim et al., 2021a; Aljazzar et al., 2022; Ammar et al., 2022; Awad et al., 2022). In this scenario, investigators are actively looking for unique microbicides with pronounced efficacy and the lowest hazardous impacts. One of the key answers for the abovementioned problems is botanical antimicrobials, which could treat VRSA strains (Karimi et al., 2018). Despite the beneficial outcomes of these plant-derived antimicrobials, more advanced nanotechnologies are urgently needed to improve their potency. Berberine (BR), an isoquinoline alkaloid, has received much attention among recent medicinal herbs with many pharmacological activities like antimicrobial (Amin et al., 1969), anti-inflammatory (Kuo et al., 2004), and antipyretic (Küpeli et al., 2002). In spite of these advantages, certain limitations accompanied its clinical usage such as its low bioavailability, and poor aqueous solubility and absorption via the gastrointestinal tract. To justify these drawbacks, the successful choice of an excellent nanocarrier delivery system is of utmost importance. Mesoporous silica nanoparticles (MPS-NPs) are one of the novel therapeutic nanocarriers with porosities, mesostructures, and tunable morphologies as well as superior functionalization and biocompatibility. This nanocarrier possesses several benefits including affordable production, dissolvability, stability, biocompatibility, and biodegradability along with the ability to release a variety of antimicrobial agents at the desired site (Fang et al., 2023). Considering all the foregoing indicators, for the first time, the current research designed *in vitro* and *in vivo* models aimed to evaluate an innovative nanocarrier delivery system combining berberine and MPS-NPs as a potential antimicrobial, anti-QS, and antivirulence agent against strong biofilm-producing and multi-virulent VRSA strains.

## 2 Materials and methods

### 2.1 Study design and characterization of *Staphylococcus aureus* strains

In the current study, we explored 95 *S. aureus* strains isolated from milk samples (n= 51) collected aseptically from mastitic cows before antimicrobials treatment and from human pus samples (n= 44) in Sharkia Governorate, Egypt. Human *S. aureus* strains were associated with infections and kindly obtained from patients admitted to University hospitals, which had attained signed informed consents of the contributing patients in the current study. Identification of *S. aureus* strains was conducted phenotypically via standard conventional phenotypic microbiological methods basing on mannitol fermentation onto mannitol salt agar, formation of characteristic golden yellow colonies, beta hemolysis onto blood agar, appearance of Gram-positive grape like clusters and biochemical reactions utilizing coagulase and catalase tests (Becker et al., 2015). Furthermore, the examined strains were molecularly confirmed via PCR investigation of nuclease (*nuc*) gene (Abd El-Hamid and Bendary, 2015). Human strains were categorized as community associated MRSA as they were isolated within 48 h of hospitalization. Moreover, community associated MRSA carriers did not meet any of the risk factors documented on hospital patients’ databases; a medical history of infection with MRSA, a history of hospitalization, residence in a long‐term care facility or surgery within 1 year prior to the date of MRSA culture or permanent medical devices or catheters at MRSA culture time. The remaining strains originated from milk samples collected from mastitic cows were categorized as livestock associated MRSA. Moreover, the recovered strains were subsequently subjected to PCR assays for confirming the existence of SCC*mec*IV and SCC*mec*V types and *pvl* gene, which are strongly associated with community and livestock-associated MRSA as described elsewhere (Zhang et al., 2005; Larsen et al., 2008).

### 2.2 Characterization of methicillin and vancomycin resistant *Staphylococcus aureus* strains

For methicillin and vancomycin resistance analyses, minimum inhibitory concentrations (MIC) of oxacillin, cefoxitin and vancomycin antibiotics (Oxoid, UK) was determined, in triplicate, against all *S. aureus* strains via broth macrodilution method in accordance with Clinical and Laboratory Standards Institute (CLSI) guidelines (CLSI, 2020). For confirming the strains as MRSA and VRSA, *S. aureus* methicillin resistance gene (*mecA*) in addition to vancomycin resistance genes (*vanA* and *vanB*) were identified via PCR assays, respectively (Niesche and Haase, 2012; Bamigboye et al., 2018).

### 2.3 Detection of biofilm producing VRSA strains

*In vitro* biofilm production among confirmed VRSA strains was detected phenotypically using Congo red agar and microtiter plate assays and genotypically via PCR detection of intercellular adhesion gene A, *icaA* (Ciftci et al., 2009; Abd El-Hamid et al., 2020). Briefly, Congo red agar medium was prepared by combining brain heart infusion broth, agar, sucrose (5%) and Congo red stain (0.8 g/L). The CRA plates were inoculated and then incubated at 37°C for 24 h, followed by storage for 48 h at room temperature. The VRSA strains were characterized as strong biofilm-producers according to their colonial morphologies as rough black colonies. The microtiter plate assay was performed briefly as following: VRSA strains were cultured onto blood agar plates and incubated at 37°C for 24h. Pure colonies were inoculated in trypticase soy broth and the suspensions were incubated at 37°C for 24 h and then diluted in fresh broth containing glucose. This dilution was further seeded on sterile 96-well tissue culture polystyrene plates that were incubated overnight for 18 h at 37°C. The plates were then rinsed three times with sterile phosphate buffered saline and dried. The attached bacteria were fixed via adding methanol for 15 minutes at room temperature. Afterwards, the plates were stained with crystal violet aqueous solution for 15 minutes at room temperature. After staining, the plates were rinsed under running water until there was no trace of stain and the bounded stain was dissolved by ethanol. The optical density was measured at 570 nm using ELISA Microplate reader (Thermo Fisher. Scientific, USA). Cut-off OD (ODc) is defined as three standard deviations above the mean OD of the negative control. Strong biofilm producers were interpreted as following: 4 × ODc < OD. Each strain was tested for biofilm production in duplicate and the assays were repeated three times.

### 2.4 Virulence genes profiling and *agr* genotyping

Amplification of four virulence genes; toxic shock syndrome toxin (*tst*), clumbing factor A (*clfA*), alpha-hemolysin (*hla*) and Panton-Valentine leukocidin (*pvl*) was done via Singleplex PCR protocols, while identification of *agr* alleles within strong biofilm producing VRSA strains was carried out through multiplex PCR assay using EmeraldAmp^®^ GT PCR Master Mix (TaKaRa Bio, Shiga, Japan). Amplification procedures were applied as pointed out formerly (Mehrotra et al., 2000; Li et al., 2019; Rasmi et al., 2022). Each PCR assay incorporated positive and negative controls comprising DNA extracted from strains of *S. aureus*; ATCC25923 and *Escherichia coli*; ATCC25922, respectively. The targeted sequences of each primer employed in PCR amplification reactions are depicted in **Table 1**.

**Table 1:** Oligonucleotide primers’ sequences used for PCR amplification assays.

Target gene	Primer sequence (5’-3’)	Reference/accession No
***nuc***	F: GCGATTGATGGTGATACGGTT R: AGCCAAGCCTTGACGAACTAAAGC	(Brakstad et al., 1992)
***mecA***	F: TCCAGATTACAACTTCACCAGG R: CCACTTCATATCTTGTAACG	(Niesche and Haase, 2012)
***vanA***	F: GGGAAAACGACAATTGC R: GTACAATGCCGTTA	(Bamigboye et al., 2018)
***vanB***	F: TCTGTTTGAATTGTCTGGTAT R: GACCTCGTTTAGAACGATG	(Bamigboye et al., 2018)
***icaA***	CCTAACTAACGAAAGGTAG AAGATATAGCGATAAGTGC	(Ciftci et al., 2009)
***tst***	F: ACCCCTGTTCCCTTATCATC R: TTTTCAGTATTTGTAACGCC	(Mehrotra et al., 2000)
***clfA***	F: ATTGGCGTGGCTTCAGTGCT R: CGTTTCTTCCGTAGTTGCATTTG	(Li et al., 2019)
***hla***	F: CTGATTACTATCCAAGAAATTCGATTG R: CTTTCCAGCCTACTTTTTTATCAGT	(Rahman et al., 2018)
***pvl***	F: ATCATTAGGTAAAATGTCTGGACATGATCCA R: GCATCAAGTGTATTGGATAGCAAAAGC	(McClure et al., 2006)
***agrI***	F: ATGCACATGGTGCACATGC R: GTCACAAGTACTATAAGCTGCGAT	(Rushdy et al., 2007; Abd El-Hamid and Bendary, 2013)
***agrII***	F:ATGCACATGGTGCACATGC R:TATTACTAATTGAAAAGTGGCCATAGC	(Rushdy et al., 2007; Abd El-Hamid and Bendary, 2013)
***agrIII***	F:ATGCACATGGTGCACATGC R:GTAATGTAATAGCTTGTATAATAATACCCAG	(Rushdy et al., 2007; Abd El-Hamid and Bendary, 2013)
***agrIV***	F:ATGCACATGGTGCACATGC R:CGATAATGCCGTAATACCCG	(Rushdy et al., 2007; Abd El-Hamid and Bendary, 2013)
***16S rRNA***	F:GTGGAGGGTCATTGGA R:CGTTTACGGCGTGGACT	(Abd El-Hamid et al., 2020)
***IL-1β***	F-TGACAGACCCCAAAAGATTAAGG R-CTCATCTGGACAGCCCAAGTC	NM_031512.2
***IL-6***	F-CCACCAGGAACGAAAGTCAAC R-TTGCGGAGAGAAACTTCATAGCT	NM_012589.2
***TNF-a***	F-CAGCCGATTTGCCATTTCA R-AGGGCTCTTGATGGCAGAGA	L19123.1
***BAX***	F-CAAGAAGCTGAGCGAGTGTCT R-CAATCATCCTCTGCAGCTCCATATT	NM_017059
***iNOS***	F-ACCTTCCGGGCAGCCTGTGA R-CAAGGAGGGTGGTGCGGCTG	NM_ 012611
***COX-2***	F-GCTCAGCC ATACAGCAAATCC R-GGGAGTCGGGCAAT CATCAG	NM_017232
***caspase-3***	F-GCAGCTAACCTCAGAGAGACATTC R-ACGAGTAAGGTCATTTTTATTCCTGACTT	NM_012922
***GAPDH***	F-TGCTGGTGCTGAGTATGTCG R-TTGAGAGCAATGCCAGCC	NM_017008

*nuc*, nuclease; *mecA*, methicillin resistance gene; *van*, vancomycin resistance gene; *icaA*, intercellular adhesion gene A; *tst*, toxic shock syndrome toxin; *clfA*, clumbing factor A; *hla*, alpha-hemolysin; *pvl*, Panton-Valentine leukocidin; *agr*, accessory gene regulator; *16S rRNA*, 16S ribosomal ribonucleic acid; *IL*, interleukin; *TNF-a*, tumor necrosis factor-alpha; *BAX*, Bcl-2-associated X protein; *iNOS*, inducible nitric oxide synthase; *COX-2*, cyclooxygenase-2; *GAPDH*, glyceraldehyde 3-phosphate dehydrogenase; F, forward; R, reverse.

### 2.5 Preparation, characterization and release of berberine conjugated MPS-NPs

**Figure 1:**
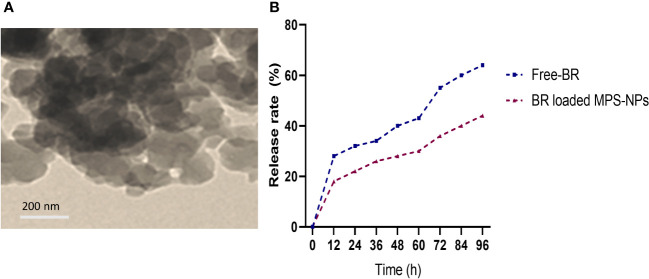
Scanning electron microscopy characterization **(A)** and release **(B)** of berberine loaded mesoporous silica nanoparticles.

Berberine (98%) was purchased from Sigma-Aldrich Co. (St Louis, MO, USA) and the preparation of MPS-NPs was done as previously described by (Alandiyjany et al., 2022). The loading of BR into MPS-NPs was carried out using the method of solvent evaporation (Nafisi et al., 2018). Briefly, 10 ​mg of BR was dissolved in methanol, then MPS-NPs was added to BR by 1:4 ratio. After that, the prepared combination was sonicated for 10 ​min away from light, stirred at 200 ​rpm for 24 ​h and the solvent was evaporated. The newly formed BR loaded MPS-NPs were separated via centrifugation for 15 ​min at 10000 ​rpm, washed to eliminate any Free-BR and dried at 37°C for 24 ​h. Characterization of prepared BR loaded MPS-NPs was determined by scanning electron microscopy (SEM, **Figure 1A**). Moreover, a dialysis bag (Molecular weight =12 kDa) was used to explore the *in vitro* release rate of BR from MPS-NPs against phosphate buffered saline at pH=7.6 and 37°C over 0, 12, 24, 36, 48, 60, 72, 84 and 96 h (**Figure 1B**).

### 2.6 Berberine conjugated MPS-NPs efficacy on strong biofilm producing and multi-virulent VRSA strains

#### 2.6.1 *In vitro* assays

##### 2.6.1.1 Antibacterial activities of Free-BR and BR loaded MPS-NPs

The antibacterial activities of Free-BR and prepared BR loaded MPS-NPs against strong biofilm producing and multi-virulent VRSA strains embracing all explored virulence genes; *icaA*, *tst, clfA, hla and pvl* utilizing the agar well diffusion (Yadav et al., 2015) and broth microdilution (Ammar et al., 2021b) methods were assessed in triplicate. The potency of tested compounds was affirmed via distinguishing largest inhibition zone diameters and lowest values of MIC.

##### 2.6.1.2 Antibiofilm activities of Free-BR and BR loaded MPS-NPs

A quantitative assessment of the antibiofilm activity of Free-BR and BR loaded MPS-NPs against the tested strains was performed using a microtiter plate assay as described earlier (Abd El-Hamid et al., 2020). The percentage of biofilm inhibition for each tested compound was calculated using the formula pronounced previously (Lopes et al., 2017). Briefly, wells of microplates were filled with VRSA suspensions in trypticase soy broth supplemented with glucose. Afterwards, the tested Free-BR and BR loaded MPS-NPs were added, at their sub inhibitory concentrations (SICs), to each well and the plates were then incubated at 37°C for 24 h. After incubation, planktonic cells were removed and the microplate wells were washed with sterile phosphate buffered saline to remove unbound planktonic cells and the plates were then air-dried. The biofilm was stained with crystal violet solution for 15 minutes, solubilized by ethanol and the absorbance was read at 570 nm using ELISA Microplate reader (Thermo Fisher. Scientific, USA). Suspensions without adding Free-BR or BR loaded MPS-NPs were tested as untreated controls. The percentage of biofilm inhibition was calculated using the following formula: percentage of inhibition = [(control OD _570 nm_ – treated OD _570nm_)/control OD _570 nm_] × 100.

##### 2.6.1.3 Modulation of virulence and *agr* genes expression

A SYBR Green quantitative reverse transcriptase polymerase chain reaction (qRT-PCR) approach (Ammar et al., 2021b) was used for determining the efficacy of Free-BR and prepared berberine conjugated MPS-NPs SICs on virulence and *agr* genes expression among strong biofilm producing and multi-virulent VRSA strains. The QIAamp RNease Mini Kit (Qiagen, USA) was employed to extract RNA from Free-BR and prepared berberine conjugated MPS-NPs treated or non-treated bacterial cells. In Stratagene real-time PCR system (MX3005P; Thermo Fisher scientific, USA), mRNA expression levels of target genes were estimated, in triplicate, utilizing their corresponding primers (**Table 1**) and the kits of QuantiTect SYBR Green PCR Master Mix (2X, Qiagen, USA). For each amplification run, 16S ribosomal ribonucleic acid (*16S rRNA*) gene was employed as a house keeping gene (Abd El-Hamid et al., 2020). For assessing qRT-PCR specificity, DNAs’ melting curves of PCR fragments were generated. Quantification of mRNA expression levels of target genes in strains exposed to Free-BR and prepared BR loaded MPS-NPs comparing with the non-exposed ones was detected utilizing the 2^-ΔΔCt^ equation (Livak and Schmittgen, 2001).

#### 2.6.2 *In vivo* model

##### 2.6.2.1 Experimental design

All experimental techniques were reviewed and approved by Animal Ethics Review Committee of Suez Canal University (AERC-SCU), Egypt; reference number, AERC-SCU 2023068. A total of 60 male mice aged 6–8 weeks and with an average weight of 22.3 g ± 0.5 were obtained from the farm of Faculty of Veterinary Medicine, Zagazig University. Mice were housed in standard environmental conditions; controlled temperature (21 ± 1°C) and humidity (57%) and a 12:12 hour light/dark cycle, fed ad libitum and tested to be free from VRSA. The experimental mice were randomly divided into three experimental groups; VRSA challenged and non-treated and two challenged and treated ones. The challenge was carried out subcutaneously using selected strong biofilm producing and multi-virulent VRSA strain containing approximately 5x10^6^ CFUs three days apart (Elmowalid et al., 2022). Post appearance of clinical signs specific for VRSA, topical treatment was done either by Free-BR and BR loaded MPS-NPs at their SIC levels prepared in aqueous phosphate-buffered saline solutions. The treatment was initiated on the 3^rd^ day post-challenge and continued for five days. The challenged mice were kept under observation for appearance of any clinical signs.

##### 2.6.2.2 Gene expression analysis

Expression analysis of VRSA virulence and *agr* genes at 10 days post infection (dpi) and genes encoding pro-inflammatory cytokines; interleukin (IL)-6, IL-1β and tumor necrosis factor-alpha (TNF-α) and pro-apoptotic indicators; Bcl-2-associated X (BAX), inducible nitric oxide synthase (iNOS), cyclooxygenase-2 (COX-2) and caspase-3 at 5 and 10 dpi were further estimated as previously detailed in section 2.6.1.3 utilizing glyceraldehyde 3-phosphate dehydrogenase (*GAPDH*) as a housekeeping gene. The primer sequences used for expression analysis are listed in **Table 1**.

### 2.7 Statistical analysis

Impact of Free-BR and BR loaded MPS-NPs on experimental tested parameters following VRSA challenge was analyzed via one-way analysis of variance (ANOVA) of SPSS software (Version 11.0; SPSS Inc., Chicago, IL, USA) and Tukey’s tests. Variations among means were statistically significant at *p* < 0.05. All graphs were made using GraphPad Prism software Version 8 (San Diego, CA, USA).

## 3 Results

### 3.1 Categorization of MRSA and VRSA strains and VRSA biofilm producers

**Figure 2:**
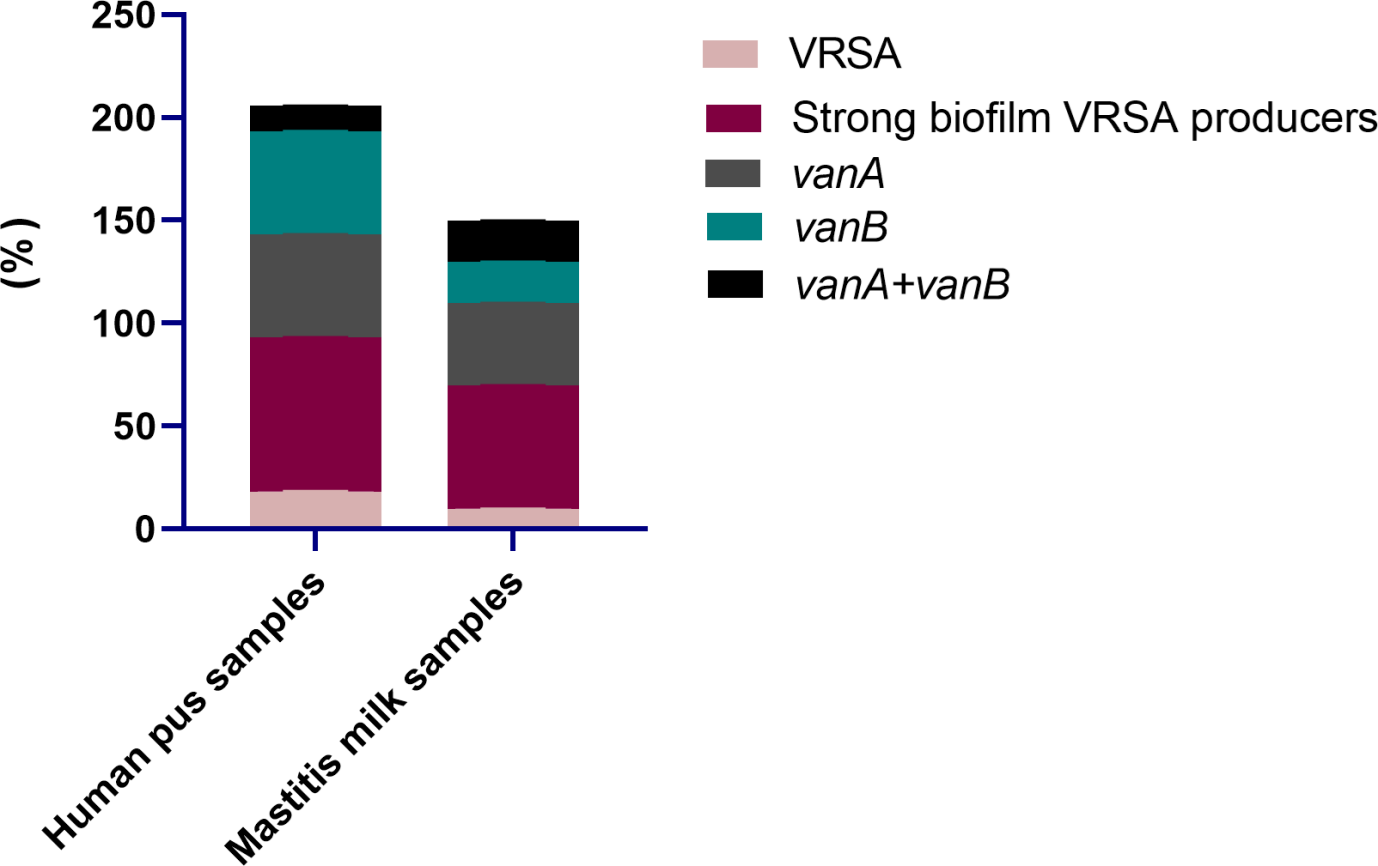
Distribution of vancomycin resistant *Staphylococcus aureus* (VRSA), strong biofilm VRSA producers and *van* genes in human pus and mastitis milk samples.

All 95 *S. aureus* strains originating from bovine and human sources were characterized phenotypically using standard microbiological tests, and genotypically via PCR amplifications of *nuc* gene. Moreover, all strains were phenotypically resistant to oxacillin and cefoxitin and were positive for *mecA* gene; thus, they were confirmed as MRSA. With regard to the epidemiological criteria, all the investigated community and livestock-associated MRSA strains harbored *pvl* gene and contained SCC*mec* types IV (72.7 and 60.8%) and V (27.3 and 39.2%) elements, respectively. Notably, vancomycin resistance with MIC values ranging from 64-1024 μg/mL was observed in 13 *S. aureus* strains (13.7%) being classified as VRSA; 18.2% (8/44) and 9.8% (5/51) from human and animal origins, respectively (**Figure 2**). PCR amplification of *van* genes among 13 VRSA strains revealed the presence of *vanA* and *vanB* genes in 6 and 5 strains (46.2 and 38.5%, respectively), and they were both identified in 2 strains (15.4%). Out of 13 VRSA strains, 9 (69.2%) were identified phenotypically as strong biofilm producers using Congo red agar and microtiter plate assays, and they were all positive for *icaA* gene. Interestingly, human samples possessed higher percentages of strong biofilm-forming VRSA (75%) than animal ones (60%, **Figure 2**).

### 3.2 Virulence gene profiling and *agr* genotyping

**Figure 3:**
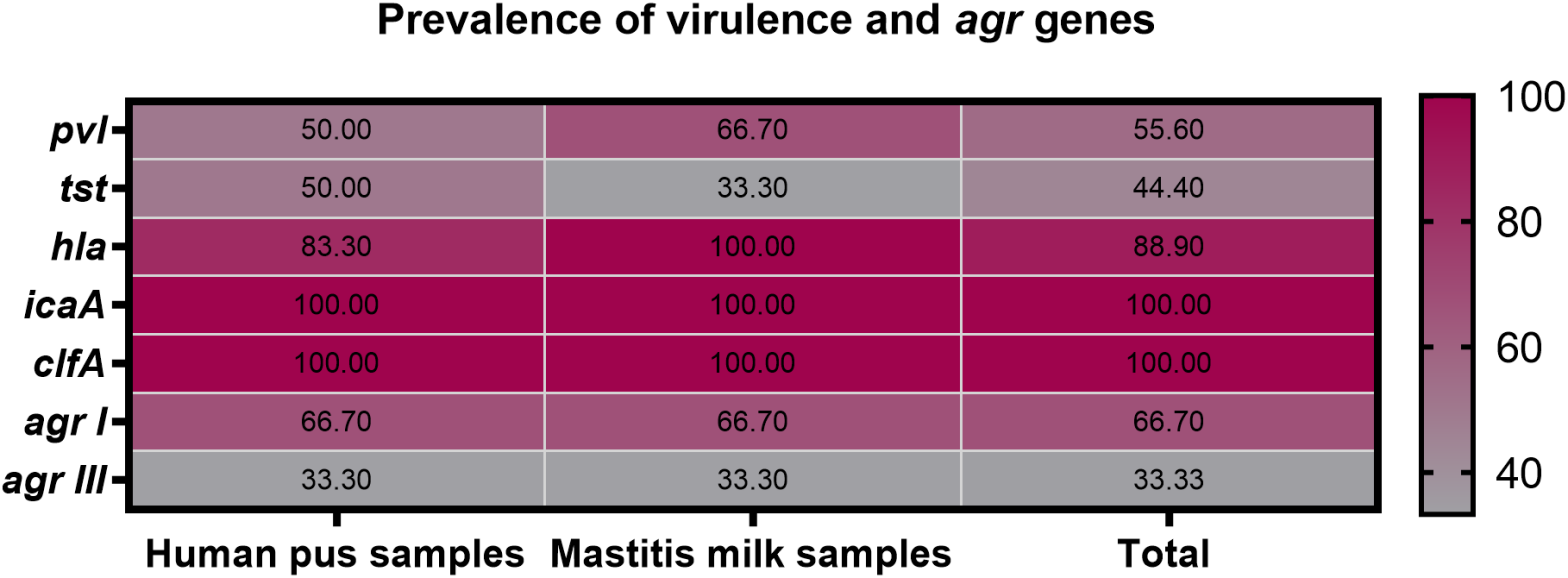
Prevalence of *icaA*, *tst*, *clfA*, *hla*, *pvl* and *agr* genes among strong biofilm producing VRSA strains recovered from human pus and mastitis milk samples.

All 9 strong biofilm-producing VRSA strains were positive for *clfA* gene (100%), while 8 (88.9%), 5 (55.6%), and 4 (44.4%) were positive for *hla*, *pvl* and *tst* genes, respectively (**Figure 3**). Moreover, *hla* and *pvl* genes prevailed among VRSA strains isolated from animal (100 and 66.7%) compared to human (83.3 and 50%) origins, respectively. Meanwhile, the prominent occurrence rate for *tst* gene was observed in human strains (50%) rather than animal ones (33.3%). There were no significant differences between VRSA strains isolated from human and animal origins regarding the distribution of virulence and *agr* genes (*P* = 0.924). Interestingly, all 9 strong biofilm-producing VRSA strains possessed a minimum of two virulence genes embracing *clfA* and *icaA*, and 8 strains were multi-virulent (88.9%) with three or more examined virulence genes. Among the recovered VRSA strains, four virulence gene profiling was identified with 44.4% of these strains possessing all five virulence genes (*icaA*, *tst*, *clfA*, *hla*, and *pvl*). Regarding *agr* genotyping, *agr* I predominated among human and animal VRSA strains (66.7% each), followed by *agr* III (33.3% each); meanwhile, none of the strains was positive for *agr* types II and IV (**Figure 3**).

### 3.3 *In vitro* impact of Free-BR and BR-loaded MPS-NPs on strong biofilm-producing and multi-virulent VRSA strains

#### 3.3.1 Antimicrobial activities of Free-BR and BR-loaded MPS-NPs

The antimicrobial activities of Free-BR and prepared BR-loaded MPS-NPs were detected on four strong biofilm-producing and multi-virulent VRSA strains harboring all five virulence genes. Excellent antimicrobial properties of Free-BR and BR-loaded MPS-NPs were found with more pronounced efficacy for the newly formulated conjugate than Free-BR against investigated strains as proved by diameters of inhibition zones of 25-30 and 20-23 mm, respectively. More pronounced VRSA growth inhibition was detected using BR-loaded MPS-NPs than Free-BR with MIC values of 0.125-0.5 and 0.5-2 μg/mL, respectively (**Table 2**).

**Table 2:** Antimicrobial and antibiofilm activities of BR-loaded MPS-NPs and FB against strong biofilm producing and multi-virulent VRSA strains.

VRSA tested strain	Antimicrobial activity				Anti-biofilm activity	
	Zone Diameter (mm)		MIC (ug/mL)		Inhibitory capacity (%)	
	BR-loaded MPS-NPs	Free-BR	BR-loaded MPS-NPs	Free-BR	BR-loaded MPS-NPs	Free-BR
**VRSA I**	27	21	0.25	1	99.76	99.27
**VRSA II**	29	22	0.125	1	99.84	99.16
**VRSA III**	30	23	0.125	0.5	99.97	99.43
**VRSA IV**	25	20	0.5	2	99.86	99.52

VRSA, vancomycin resistant *Staphylococcus aureus*; BR-loaded MPS-NPs, berberine-mesoporous silica nanoparticles; Free-BR, free berberine; MIC, minimum inhibitory concentration; SIC; sub inhibitory concentration.

#### 3.3.2 Antibiofilm activities of Free-BR and BR-loaded MPS-NPs

Analyzing the quantitative phenotypic detection of biofilm post exposure to Free-BR and BR-loaded MPS-NPs using the microtiter plate assay revealed prominent decrease in the capacity of all strong biofilm-producing and multi-virulent VRSA strains post exposure to the examined compounds when compared with the untreated ones with inhibitory capacity percentage up to 99.97%. Of note, BR-loaded MPS-NPs exhibited more pronounced antibiofilm capacity than Free-BR as evidenced by inhibitory capacity percentage ranges of 99.76 to 99.97% for BR-loaded MPS-NPs and 99.16 to 99.52% for Free-BR (**Table 2**). There was a significant difference between BR-loaded MPS-NPs and FB in their inhibitory capacity percentage (*P* < 0.001).

#### 3.3.3 Gene expression analysis post exposure to Free-BR and BR-loaded MPS-NPs

**Figure 4:**
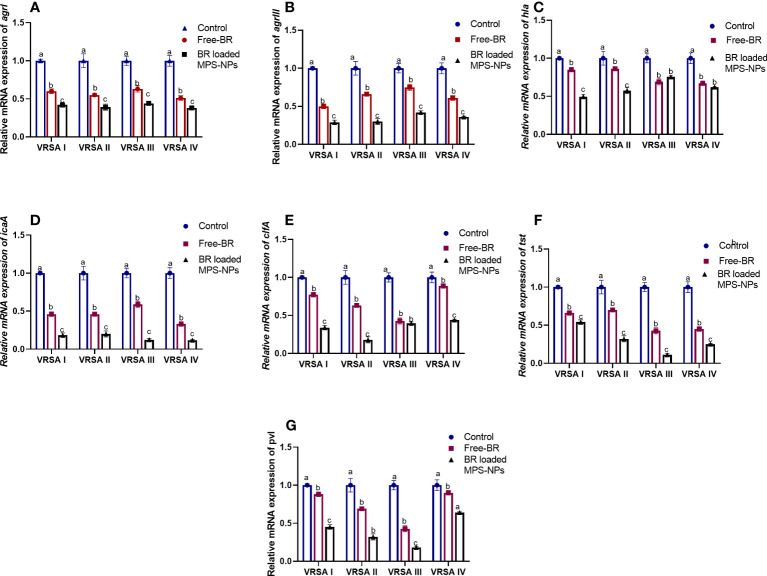
Relative mRNA expression levels of *agrI* **(A)**, *agrIII* **(B)**, *hla* **(C)**, *icaA* **(D)**, *clfA* **(E)**, *tst* **(F)** and *pvl* **(G)** genes expression among four strong biofilm producing and multi-virulent vancomycin-resistant *Staphylococcus aureus* (VRSA) strains exposed to SICs of Free berberine (Free-BR) and prepared berberine conjugated mesoporous silica nanoparticles (BR loaded MPS-NPs) comparing with unexposed ones (control) with an estimated value of 1. The values expressed the means of three experiments ± standard error (error bar). ^a–c^ Means within the same column carrying different superscripts are significantly different at *p* < 0.05.

The qRT-PCR technique was utilized to evaluate the efficacy of Free-BR and prepared BR-loaded MPS-NPs on the mRNA expression of virulence (*icaA*, *tst*, *clfA*, *hla*, and *pvl*) and *agr* genes in the four strong biofilm-producing and multi-virulent VRSA strains. The transcript levels of *agr* and virulence genes were prominently (*p >* 0.05) reduced following VRSA strains exposure to the explored compounds (up to 0.29 and 0.11-fold, respectively). Notably, the broad-spectrum antivirulence activity of BR-loaded MPS-NPs was achieved via striking downregulated expression levels of VRSA target genes (up to 0.11-fold). Additionally, Free-BR decreased examined genes expression (up to 0.33-fold). Remarkably, Free-BR and formulated BR-loaded MPS-NPs displayed higher suppression levels for *agr*, *clfA*, *pvl*, *tst*, *hla*, and *icaA* genes (decreased by 0.50 and 0.29, 0.42, and 0.17, 0.43 and 0.18, 0.42 and 0.11, 0.67 and 0.49 and 0.33 and 0.11-fold, respectively (**Figure 4**).

### 3.4 *In vivo* impact of Free-BR and BR-loaded MPS-NPs

#### 3.4.1 Pro-inflammatory and pro-apoptotic gene expression

**Figure 5:**
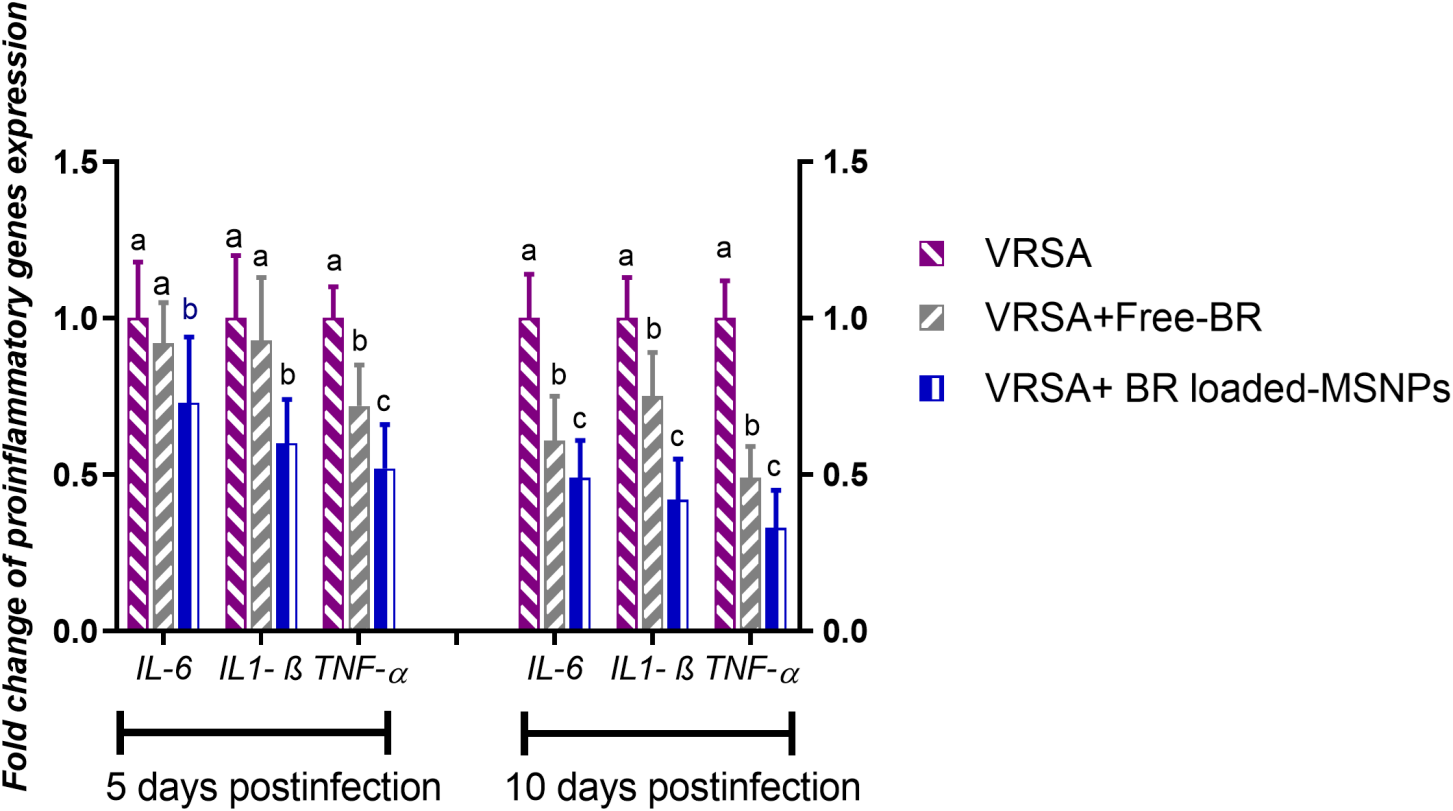
Relative mRNA expression levels of pro-inflammatory cytokine genes; interleukin (*IL)-* 6, *IL-1β* and tumor necrosis factor-alpha (*TNF-α*) in vancomycin resistant *Staphylococcus aureus* (VRSA) challenged mice and VRSA challenged mice and treated with Free-BR or BR loaded MPS-NPs at 5 and 10 days post infection (dpi). VRSA: mice challenged with vancomycin resistant *Staphylococcus aureus*; VRSA+Free-BR: mice challenged with vancomycin resistant *Staphylococcus aureus* and treated with Free berberine; VRSA+BR loaded MPS-NPs: mice challenged with vancomycin resistant *Staphylococcus aureus* and treated with berberine-loaded mesoporous silica nanoparticles. ^a–c^ Means within the same column carrying different superscripts are significantly different at *p* < 0.05.

**Figure 6:**
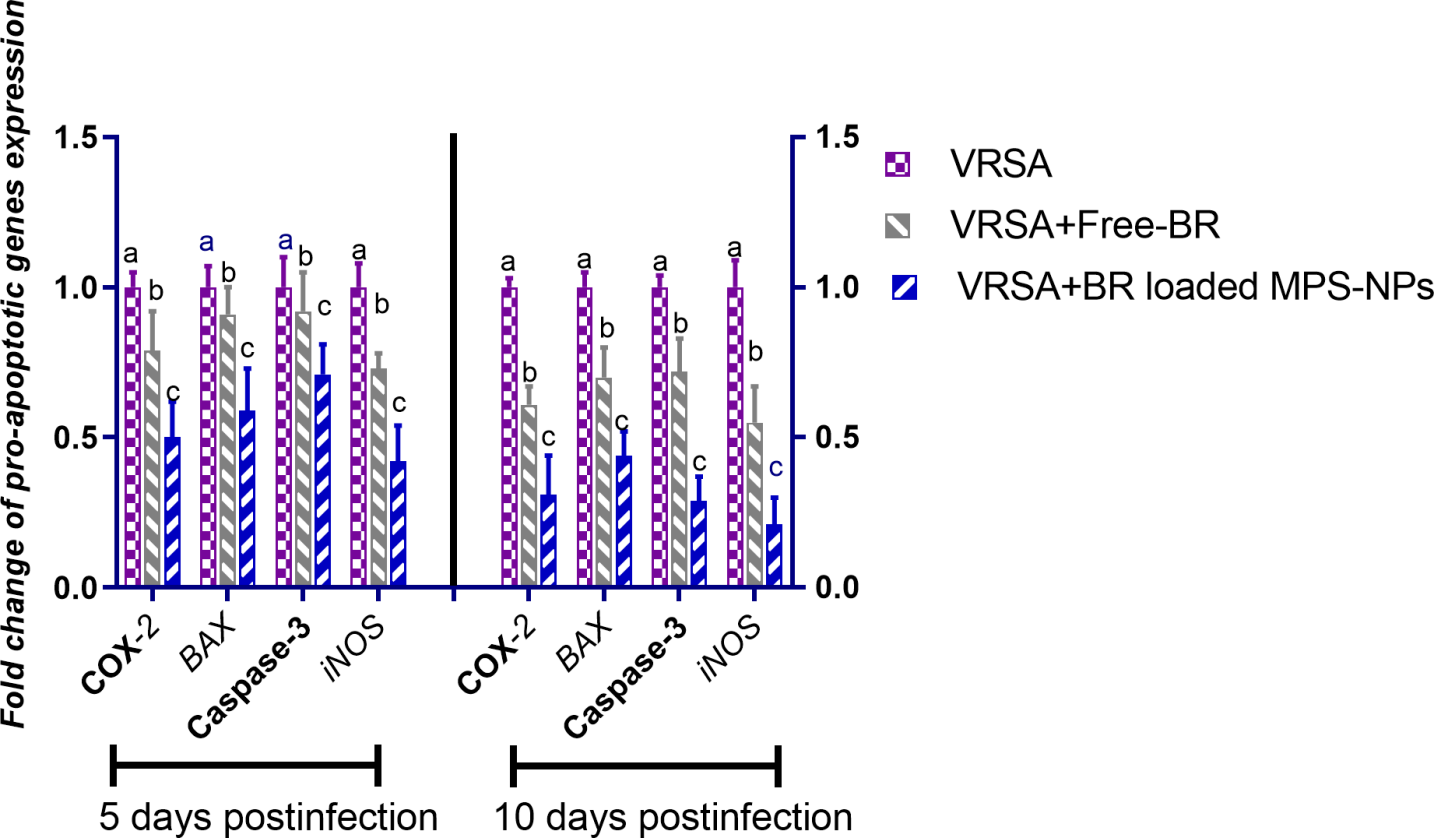
Relative mRNA expression levels of pro-apoptotic genes; cyclooxygenase-2 (*COX-2*), caspase-3, inducible nitric oxide synthase (*iNOS*), and Bcl-2-associated X protein (*BAX*) in vancomycin resistant *Staphylococcus aureus* (VRSA) challenged mice and VRSA challenged mice and treated with Free-BR or BR loaded MPS-NPs at 5 and 10 days post infection (dpi). VRSA: mice challenged with vancomycin resistant *Staphylococcus aureus*;VRSA+Free-BR: mice challenged with vancomycin resistant *Staphylococcus aureus* and treated with Free berberine; VRSA+BR loaded MPS-NPs: mice challenged with vancomycin resistant *Staphylococcus aureus* and treated with berberine-loaded mesoporous silica nanoparticles. ^a–c^ Means within the same column carrying different superscripts are significantly different at *p* < 0.05.

Regarding gene expression analysis of IL-6, IL-1β, and TNF-α pro-inflammatory cytokines, marked downregulation was noticed at 10 dpi in VRSA-challenged groups treated with either Free-BR or BR-loaded MPS-NPs. At 5 dpi, there was no significance difference in the expression levels of *IL-6*, *IL-1β* genes between VRSA-challenged group and VRSA-challenged group treated with Free-BR. The most prominent downregulation of *IL-6*, *IL-1β*, and *TNF-*α genes was detected in the VRSA-challenged group treated with BR-loaded MPS-NPs (0.49, 0.42, and 0.33-fold change, respectively) at 10 dpi (**Figure 5**). The expression levels of pro-apoptotic genes (*BAX*, *iNOS*, *COX-2*, and caspase-3) at 5 and 10 dpi are illustrated in **Figure 6**. At 5 dpi, all investigated apoptotic genes were significantly downregulated in VRSA-challenged groups treated either with Free-BR or BR-loaded MPS-NPs unlike the untreated VRSA-challenged group. At 10 dpi, notable downregulation of *BAX*, *iNOS*, *COX-2*, and caspase-3 genes was recorded in the VRSA-challenged group treated with BR- loaded MPS-NPs, followed by that treated with Free-BR.

#### 3.4.2 Antivirulence activities of Free-BR and BR-loaded MPS-NPs

**Figure 7:**
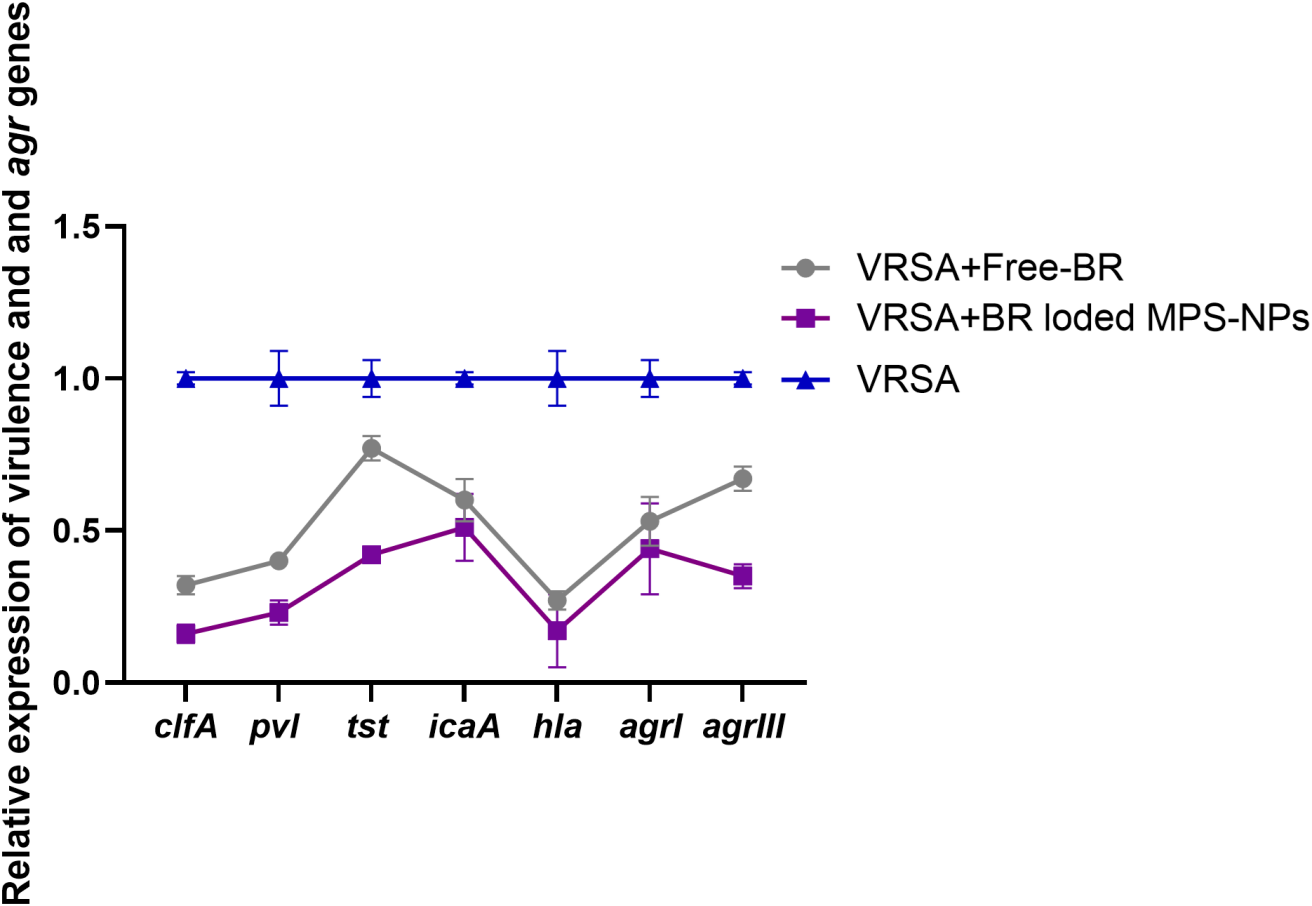
Relative mRNA expression levels of *agrI*, *agrIII*, *hla*, *icaA*, *clfA*, *tst* and *pvl* genes expression at 10 days post experimental infection with strong biofilm producing and multi-virulent vancomycin resistant *Staphylococcus aureus* (VRSA) strain in VRSA challenged mice and VRSA challenged mice and treated with Free-BR or BR loaded MPS-NPs. VRSA: mice challenged with vancomycin resistant *Staphylococcus aureus*; VRSA+Free-BR: mice challenged with vancomycin resistant *Staphylococcus aureus* and treated with Free berberine; VRSA+BR loaded MPS-NPs: mice challenged with vancomycin resistant *Staphylococcus aureus* and treated with berberine-loaded mesoporous silica nanoparticles. The values expressed the mean of three independent experiments ± standard error (error bar).

After the observation period of VRSA-challenged and VRSA-challenged and treated mice, slight clinical signs in the form of skin lesions were realized in mice treated with Free-BR and BR-loaded MPS-NPs compared to severe development of skin abscesses and reduction in mice activity in the VRSA-challenged non-treated group. Investigating virulence and *agr* gene expression revealed most prominent downregulation in the VRSA-challenged and BR-loaded MPS-NPs-treated group (up to 0.16 and 0.35-fold change, respectively), which came concurrently with the suppression of the severity of clinical signs in this group at 10 dpi. Notably, *hla* gene was the most downregulated gene in the BR-loaded MPS-NPs treated group (0.16-fold change) (**Figure 7**).

## 4 Discussion

In recent years, increasing rates of VRSA strains have spurred more research toward novel antimicrobial agents (Thati et al., 2011). Accordingly, natural antimicrobial substances have been explored as candidates for controlling VRSA (Yu et al., 2005). Among these agents, berberine alkaloids are considered to be major components with broad-spectrum antimicrobial efficacy against a variety of bacterial species (Jiao-Yang et al., 2014). Therefore, our aim was to evaluate the antimicrobial, antibiofilm, anti-quorum sensing, and antivirulence activities of an innovative nanocarrier delivery system combining berberine and MPS-NPs against strong biofilm-producing and multi-virulent VRSA strains. In the present study, all *S. aureus* strains were characterized phenotypically via conventional microbiological tests, and genotypically via PCR amplifications of *nuc* gene as reported elsewhere (Ammar et al., 2016) (Ammar et al., 2022).

Remarkably, the differences in the patterns of antimicrobial-resistant *S. aureus* isolates among various districts are attributed to the variations in the prescribed antimicrobial agents (Ammar et al., 2021a). Our results revealed that all *S. aureus* strains (100%) were resistant to oxacillin, which was estimated to be higher than the findings of previous researches performed in Cameroon (74%) (Bissong et al., 2020), South Africa (65.1%) (Pekana and Green, 2018), and Egypt (52.6%) (Abd El-Hamid and Bendary, 2015). Meanwhile, a lower resistance rate of *S. aureus* strains was detected against vancomycin (13.7%), which coordinated with the results of an Egyptian previous study, 15.8% (Abd El-Hamid et al., 2020). Therefore, opening new avenues for improving the efficacy of using natural alternative phytogenics-based therapy in animals and humans is urgently needed to mitigate the spread of antimicrobial resistance (Elmowalid et al., 2019; Ibrahim et al., 2019; Bendary et al., 2020; Ibrahim et al., 2021a; Ibrahim et al., 2021b; Ibrahim et al., 2021c; Awad et al., 2022; Hashem et al., 2022; Ibrahim et al., 2022a; Ibrahim et al., 2022b). Moreover, 69.2% of VRSA strains were identified phenotypically as strong biofilm producers using Congo red agar and microtiter plate assays, and they were all positive for *icaA* gene. These findings are in line with a previous study carried out in Egypt (Abd El-Hamid et al., 2020), where 36.7% of VRSA isolates were biofilm producers and had *icaA* gene.

Herein, all recovered MRSA strains had *clfa* gene as proven previously (Li et al., 2019). Moreover, a higher proportion of MRSA strains possessed *hla* gene (88.9%), which exceeded the findings of a previous study carried out in Egypt (37.3%) (Rasmi et al., 2022). Interestingly, 88.9% of our VRSA strains were multi-virulent with three or more investigated virulence genes. These outcomes are lower than those of previous researches (100%) in Brazil (Cavalcante et al., 2021) and China (Ren et al., 2020). The differences in the existence of targeted virulence genes could be attributed to variations in examined sources of the collected samples as well as geographical areas (Aljazzar et al., 2022). In this study, *agr* typing revealed that all VRSA strains were assigned to two *agr* allelic groups with *agr* I predominated VRSA strains (66.7%), followed by *agr* III (33.3%); meanwhile, none of the strains was positive for *agr* types II and IV. These findings were reported in previous papers reported elsewhere (Ruzin et al., 2001; Peerayeh et al., 2009; Udo et al., 2009; Abd El-Hamid and Bendary, 2013).

Free berberine (Free-BR) and BR-loaded MPS-NPs showed excellent *in vitro* antimicrobial, antibiofilm, anti-QS, and antivirulence effectiveness at their SICs against strong biofilm-producing and multi-virulent VRSA strains with more pronounced impact detected for BR-loaded MPS-NPs. Similarly, MRSA growth and biofilm were completely suppressed after berberine exposure at sub-MIC doses (Chu et al., 2016; Zhang et al., 2020). Numerous researchers had explored berberine mechanistic action against *S. aureus* (Severina et al., 2001; Wang et al., 2008). This might be related to berberine actions that penetrate the phospholipid bilayers of the bacterial membrane (Severina et al., 2001) with a consequence of compromised cell membrane integrity through lipid fluctuation. Extensive research has been carried out on various types of nanoparticles containing berberine as potential antibiofilm agents. In a recent research in China, berberine-chitosan nanoparticles had noteworthy concentration-dependent inhibitory effect on *Candida albicans* biofilm (Lin et al., 2023). Encapsulation of berberine in liposomes successfully augmented the antibiofilm activity of berberine via enhancing its uptake in bacterial cells and this was evidenced by the excellent *in vitro* effectiveness of the developed liposomes against MRSA biofilm formation and its associated intracellular infection (Bhatia et al., 2021). Moreover, self-assembling of berberine into nanoparticles displayed a better inhibitory effect on multidrug-resistant MRSA and stronger ability for biofilm removal (Huang et al., 2020).

Recently, MPS-NPs constituted more safer and effective therapeutic delivery vehicles for natural phytogenics. Herein, the augmented antimicrobial role of BR-loaded MPS-NPs against examined VRSA strains were correlated to berberine in conjugation with MPS-NPs. Our success for the choice of MPS-NPs as vehicles to load berberine resulted in BR-loaded MPS-NPs conjugate with the following properties: attaining better performance, allowing responsive delivery concurrently, and providing a nanoplatform that cures infectious VRSA diseases synergistically while avoiding drug resistance. Moreover, MPS-NPs with admirable surface properties and porosity have proven to be attractive materials (Fang et al., 2023) and effective and safe therapeutic tools for increasing the concentration of loaded bioactive compounds in the treatment region (Koohi Moftakhari Esfahani et al., 2022).

Hypervirulent VRSA mostly disrupts the host defense via suppressing phagocytic capacity and thus accelerating infection (Diep et al., 2010). This highlighted the necessity for developing efficient natural alternative therapeutic regimens to reduce antibiotic therapeutic doses, adverse effects as well as treatment duration (Liang et al., 2014). The SICs of numerous natural antimicrobials could attenuate bacterial pathogenicity and virulence via alteration of their virulence genes (Wagle et al., 2019; Ammar et al., 2022). Our results revealed that BR-loaded MPS-NPs at their SICs had significant roles in decreasing the pathogenicity of strong biofilm-producing and multi-virulent VRSA strains through reducing the expression levels of investigated virulence genes: *icaA, tst, clfA, hla, pvl*, and *agr*. Notably, these results came alongside our *in vivo* findings on a VRSA-infected mice model with subsiding the severity of clinical signs and downregulating the expression of investigated virulence genes, especially in mice treated with BR-loaded MPS-NPs. Similarly, MRSA adhesion and intracellular invasion were notably decreased post treatment with berberine (Yu et al., 2005). Moreover, expression of MRSA cell wall hydrolysis, serine protease (Wang et al., 2020), and biofilm (Zhang et al., 2022)-associated genes were significantly repressed following treatment with berberine. A previous study demonstrated that higher doses of berberine were more effective in increasing cell membrane permeability of MRSA (Xia et al., 2022). Therefore, the augmented effectiveness of BR-loaded MPS-NPs in our study is outstanding for berberine incorporation with an effective nanocarrier system: MPS-NPs. In the same context, loading drugs with silica NPs had significant roles on suppression of virulence genes and subsequent pathogenesis of MRSA superbug (Tan et al., 2018).

In our experiment, the VRSA challenge induced significant upregulation of pro-inflammatory cytokines, which indicated excessive immune response post-infection causing tissue damage. During infection, several pathogens have developed complicated strategies to modify or disrupt host cell death programs (Lamkanfi and Dixit, 2010). *Staphylococcus aureus* can trigger programmed cell death to cause infection and invade host tissues via employing many virulence factors with powerful toxigenic or immunomodulatory properties (Zhang et al., 2017). In this manner, *S. aureus* not only escapes from host immune cell responses, but also promotes tissue injury with subsequent infiltration into deeper tissues, organs, or circulating body fluids. In accordance, VRSA infection provoked upregulated relative mRNA expression levels of pro-inflammatory genes (*IL-1β*, *TNF-α*, and *IL-6*) and apoptosis-associated genes (caspase-3, caspase-9, and *Bax*) (Shaukat et al., 2021). Moreover, BR treatment suppressed activation of splenocytes and pro-inflammatory cytokine release in staphylococcal enterotoxin B-stimulated splenocytes (Du et al., 2018). Berberine, one of the well-studied medicinal plant derivatives, has a promising anti-inflammatory role in inflammatory conditions via modifying unnecessary immune responses stimulated by many immune cells (Li et al., 2023). Herein, the excessive immune response following VRSA challenge subsided after berberine treatment. Moreover, this unnecessary immune response was improved by treatment with BR-loaded MPS-NPs. Pro-inflammatory cytokines such as TNF-α and gamma interferon induce the transcription of iNOS (Liew, 1994). Caspases play a potent role in apoptosis, and their induction takes place upon intracellular complex mechanisms accounting for pro-inflammatory cytokines maturation such as IL-1β and IL-18 (Kostura et al., 1989; Lara-Tejero et al., 2006). Therefore, they act on inflammation and innate immune host defense against microbial pathogens (Martinon and Tschopp, 2004). In this manner, *S. aureus* initiates a pro-apoptotic milieu that promotes extrinsic apoptosis in the nearest host target cells. Expression of mRNA of the inducible isoform of iNOS was induced in the spleens and kidneys of *S. aureus*-infected mice (Sasaki et al., 1998). As expected, our findings revealed upregulated mRNA levels of the pro-apoptotic genes: *BAX, COX*-2, caspase-3, and *iNOS* induced by VRSA denoting infection and promoting apoptosis. However, treatment with BR-loaded MPS-NPs significantly reduced apoptosis via downregulation of pro-apoptotic genes. The augmented properties of berberine after loading on MPS-NPs were attributed to its higher bioavailability (Li et al., 2019).

## 5 Conclusion

The current study highlighted for the first time the role of incorporating Free-BR with MPS-NPs against the alarming emergence of strong biofilm-producing and multi-virulent VRSA strains. Hence, our findings demonstrated the promising *in vitro* antimicrobial, antibiofilm, anti-QS, and anti-virulence activities of BR-loaded MPS-NPs on this threatening superbug. Our *in vivo* mice experimental model signified the potential therapeutic effect of BR-loaded MPS-NPs against VRSA infection. Thus, the current research endorses the prospective application of BR-loaded MPS-NPs as an efficient therapeutic alternative for antimicrobials for controlling multi-virulent VRSA strains.

## Data availability statement

The original contributions presented in the study are included in the article/supplementary materials, further inquiries can be directed to the corresponding author.

## Ethics statement

All experimental techniques were reviewed and approved by Animal Ethics Review Committee of Suez Canal University (AERC-SCU), Egypt; reference number, AERC-SCU 2023068. Moreover, human *S. aureus* strains were kindly obtained from patients admitted to University hospitals, which had attained signed informed consents of the contributing patients in the current study.

## Author contributions

MIA: Conceptualization, Investigation, Resources, Validation, Writing – original draft, Writing – review & editing. DI: Conceptualization, Formal analysis, Investigation, Methodology, Project administration, Software, Validation, Visualization, Writing – original draft, Writing – review & editing. STE: Conceptualization, Methodology, Validation, Writing – original draft. WMG: Data curation, Methodology, Software, Writing – review & editing. MS: Investigation, Validation, Writing – original draft. WME: Formal analysis, Methodology, Resources, Software, Writing – original draft. MAA: Funding acquisition, Visualization, Writing – original draft. AS: Formal analysis, Investigation, Validation, Writing – original draft. RMA: Data curation, Funding acquisition, Investigation, Methodology, Validation, Writing – original draft, Writing – review & editing. MA: Conceptualization, Investigation, Methodology, Writing – original draft. FMS: Methodology, Writing – original draft, Writing – review & editing. AA: Formal analysis, Investigation, Resources, Validation, Writing – original draft, Writing – review & editing. EAAM: Data curation, Formal analysis, Validation, Visualization, Writing – original draft.
